# Is There a Different Mechanism for Water Oxidation in Higher Plants?

**DOI:** 10.1021/acs.jpcb.3c03029

**Published:** 2023-07-19

**Authors:** Yu-Tian Song, Xi-Chen Li, Per E. M. Siegbahn

**Affiliations:** † College of Chemistry, Beijing Normal University, Beijing 100875, China; ‡ Department of Organic Chemistry, Arrhenius Laboratory, Stockholm University, SE-106 91 Stockholm, Sweden

## Abstract

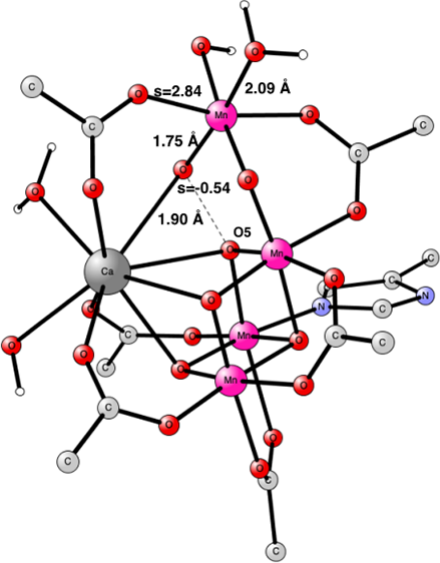

The leading mechanism for the formation of O_2_ in photosystem II (PSII) has, during the past decade, been established as the so-called oxyl–oxo mechanism. In that mechanism, O_2_ is formed from a binding between an oxygen radical (oxyl) and a bridging oxo group. For the case of higher plants, that mechanism has recently been criticized. Instead, a nucleophilic attack of an oxo group on a five-coordinated Mn(V)=O group forming O_2_ has been suggested in a so-called water-unbound (WU) mechanism. In the present study, the WU mechanism has been investigated. It is found that the WU mechanism is just a variant of a previously suggested mechanism but with a reactant and a transition state that have much higher energies. The addition of a water molecule on the empty site of the Mn(V)=O center is very exergonic and leads back to the previously suggested oxyl–oxo mechanism.

## 1 Introduction

The formation of dioxygen from water and sunlight is the most important reaction in nature for the development of higher forms of life. The key steps are performed at the oxygen-evolving center (OEC) in photosystem II. The OEC is composed of a metal cluster containing four manganese and one calcium connected by oxygen bridges. Dioxygen is evolved after absorbing four photons, going through intermediates termed S states, where O_2_ is formed in S_4_. The steps involve release of electrons to the oxidant P_680_^+^ in the reaction center and release of protons to the lumen. The S_3_ state is the last state observed before dioxygen formation and has been structurally and spectroscopically characterized.^1−5^ There is nearly perfect agreement between these experiments and the prior theoretical predictions.^6−9^ It is generally accepted that the oxidation state of S_3_ has four Mn(IV).

In the leading mechanism for dioxygen formation in S_4_, a bound oxygen radical is formed. From S_3_, the formation of S_4_ is rather strongly uphill in energy, and S_4_ has therefore never been directly observed. Theoretical model calculations have shown that O_2_ is formed from the binding of the oxygen radical with a bridging oxo group, termed the oxyl–oxo mechanism.^6−9^

**Figure 1:**
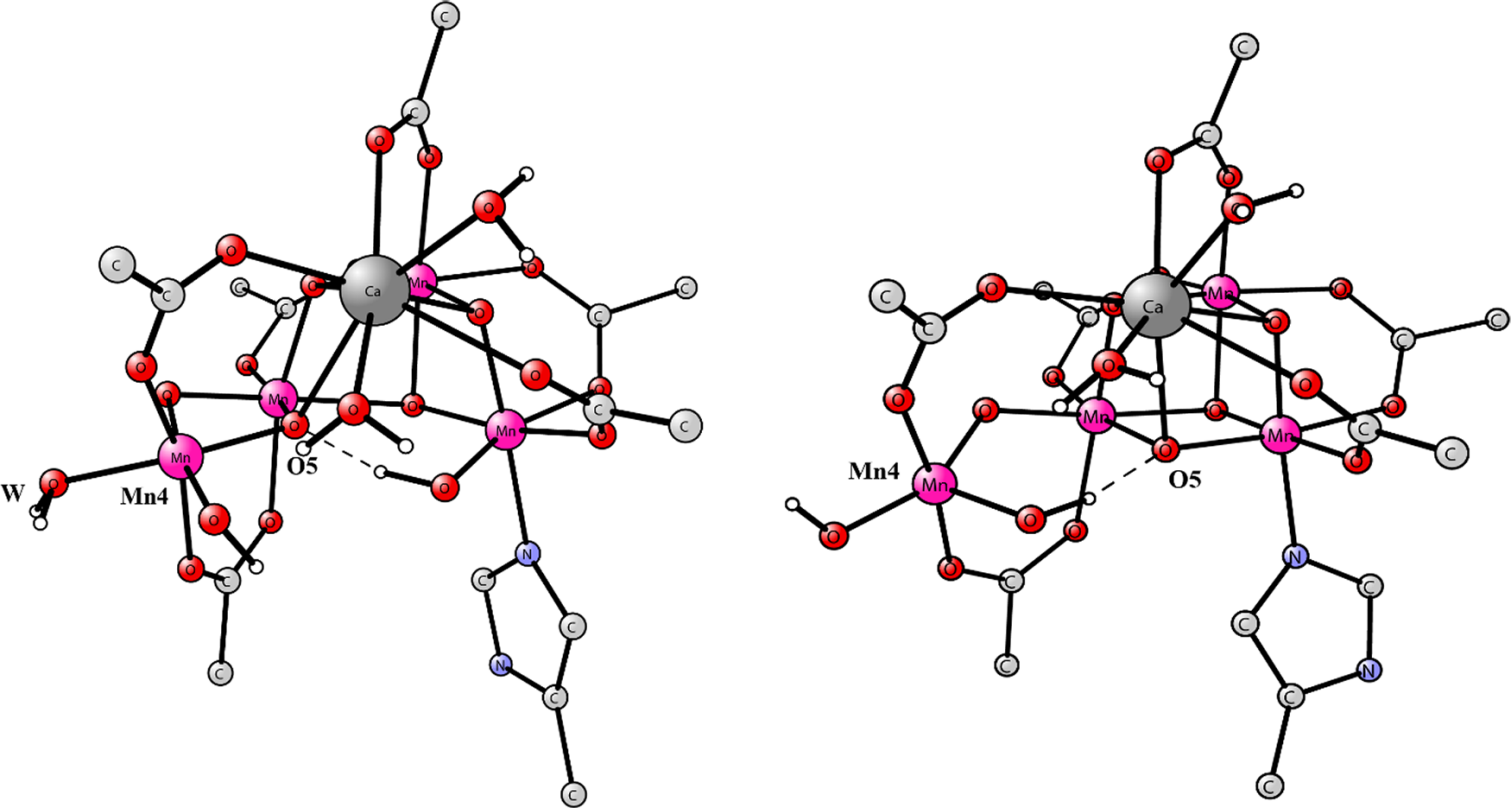
S_3_ state with an open cubane^9^ to the left and the one with a closed cubane and a missing water suggested by Pantazis and co-workers to the right.^10^

In a series of papers by Pantazis et al., the leading mechanism has been questioned for the case of higher plants.^10−13^ The starting point was an EPR observation of an S = 6 state of S_3_, observed by EPR for higher plants, which was assigned as a closed cubane structure, rather than the open cubane X-FEL structures observed for blue-green algae, see Figure 1. The terminology relates to the formation of three Mn-centers and calcium, bound by oxygens in a closed cube. In the open cubane, one of the oxygens (O5) goes out of the cube and instead forms a bond to the fourth manganese. The new suggestion with a closed cubane was made by model calculations analyzing the EPR spectrum. An additional feature is that a water, previously suggested as entering in S_3_, is no longer present. That meant that the outer, dangling Mn4 becomes coordinately unsaturated. The new mechanism was therefore termed “the water-unbound mechanism” (here termed the water-unbound (WU) mechanism). The water derived ligands on Mn4 are then two hydroxides.

In a very recent paper by Messinger and co-workers, a new mechanism for dioxygen formation in higher plants was suggested. The starting point was the earlier assignment of the S_3_ state as a closed cubane in the WU mechanism.^14^ To reach the new S_4_ state, an electron and a proton were removed from S_3_. The water derived ligands on the dangling manganese are then one hydroxide and one unprotonated oxygen. The most striking feature of the modeling of the mechanism used is that the negative Asp61 was removed from the model in spite of its strong hydrogen bond to a water on the dangling manganese in the earlier suggested open cubane S_4_ structure.

In the present paper, the recent mechanism by Messinger and co-workers is investigated with the methodology used during several previous studies that led to the oxyl–oxo mechanism. The present investigations indicate major problems in the recent study of the WU mechanism.

## 2 Methods

The methods used here have been described in several papers, see, for example, refs (6, 9, 16, 18). The standard B3LYP method was used for the geometries with the fraction of exact exchange equal to 20%.^19^ A LACVP* basis set was used. For the final energies, B3LYP was modified using 15% since it has been shown to generally be the best choice.^16−18^ In these calculations, a large cc-pvtz(−f) basis set was used. The cluster model^20^ used for the active site contains about 200 atoms, all described by hybrid DFT. To take account of the fact that the active site is constrained by the enzyme surrounding, some atoms in the outer part of the model were kept fixed from the X-ray structure. For details, see the SI. This procedure has been well tested over the years. In describing polarization effects from the surrounding enzyme, a standard dielectric cavity method was used.^21^ To account for dispersion effects, the D2 method was used.^22^ Approximate transition states were obtained varying the O–O bond distance. The calculations were done using the Jaguar^21^ and Gaussian^23^ programs.

The present calculations are built on the large experience obtained during the past decades. Each Mn atom has the highest possible spin. For the coupling between the Mn-spins in S_4_, which is the state studied here, it is important that the two Mn atoms involved in O–O bond formation have opposite spins.^6^ All states studied are sextets.

## 3 Results

The WU mechanism for plants by Messinger and co-workers, mentioned in the introduction, has a few characteristic features.^14^ EPR measurements for plants show that S_3_ is an S = 6 state in contrast to the case of blue-green algae which has an S = 3 state.^13^ Quantum chemical EPR calculations by Pantazis et al. identified the S = 6 state as a closed cubane with a five-coordinated Mn4, see Figure 1.^10−13^ That S_3_ state had been studied earlier in 2017, by the same methods as used here, and found to be much higher in energy than the open cubane structure.^15^ Those calculations were incidentally performed for an S = 6 state. The energy difference was found to be +18.1 kcal/mol, which has here been corrected to +14.9 kcal/mol after increasing the accuracy of the geometry optimization. The energy of +14.9 kcal/mol should be seen in relation to the normal accuracy of the methodology used, which is about 3 kcal/mol.^16^ Therefore, the suggested closed cubane structure must be ruled out as a possible S_3_ structure. In a recent study by O’Malley and co-workers, the EPR analysis of S_3_ as a closed cubane was also strongly criticized. In their new EPR analysis, an open cubane structure was instead clearly favored.^24^ An open cubane suggestion for the S = 6 state will be given below. For blue-green algae, X-FEL studies have identified S_3_ as an open cubane,^3,4^ in very close agreement with previous quantum calculations.^9^

**Figure 2:**
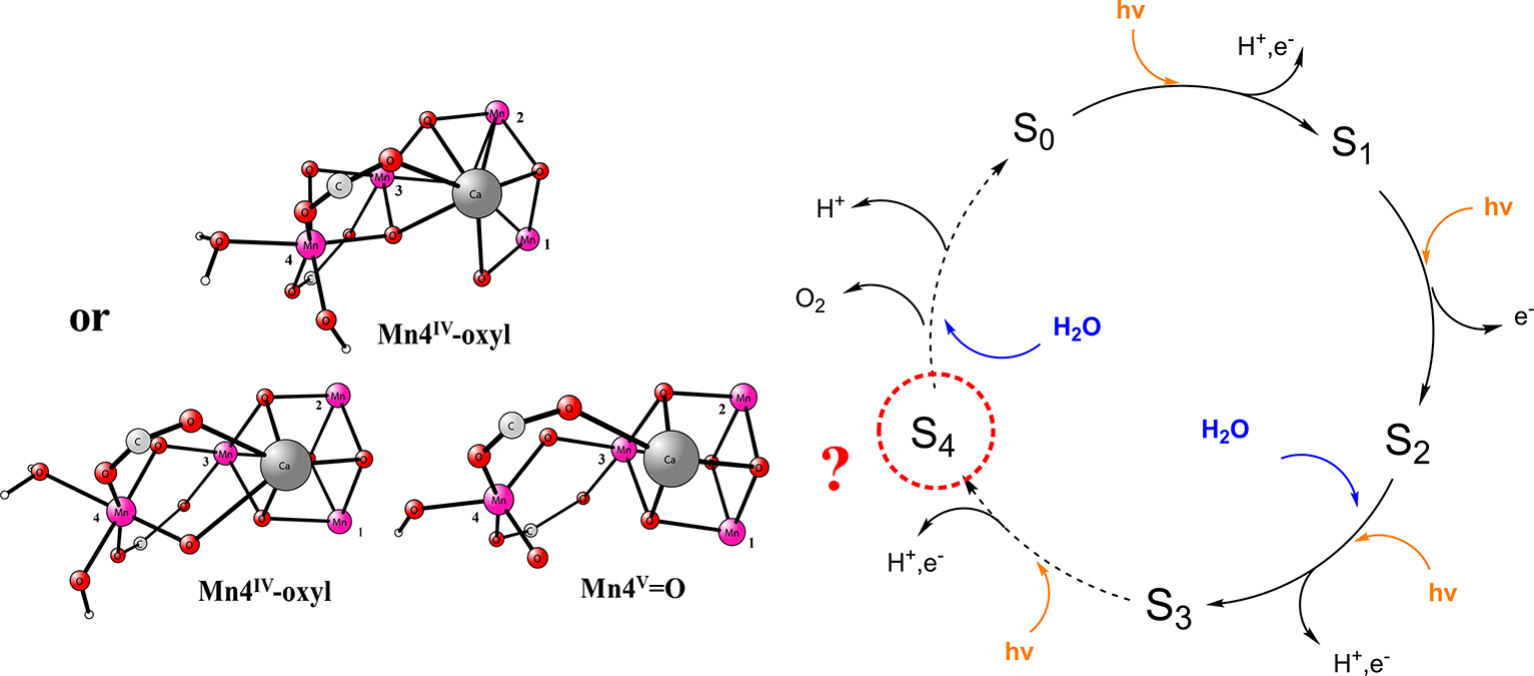
S_4_ state with an open cubane,^9^ upper figure to the left, and the one with a closed cubane, lower figure with a six-coordinated Mn4, from a previous study. The energy difference between the structures is 5.2 kcal/mol in favor of the open cubane. To the right, there is a scheme of the entire reaction cycle.

The present study is an investigation of the feasibility of the WU O–O bond formation mechanism for S_4_. A scheme of the entire reaction cycle for O_2_ formation is shown to the right in Figure 2. The closed cubane structure for S_3_ suggested by Pantazis et al. was chosen as the starting point for the WU mechanism. Therefore, by removing an electron and a proton from S_3_, a five-coordinated Mn(V)=O structure for the dangling manganese (Mn4) was obtained as a key structure for the WU S_4_ mechanism. The start of our study was an attempt to locate such a structure, starting from a closed cubane S_4_ structure found in an earlier study, see Figure 2.^25^ That structure is 5.2 kcal/mol higher than the open cubane in the same figure. It has an oxyl radical with a spin of 0.61 on a six-coordinated Mn4. Shortening the Mn–O distance from 1.76 Å for the oxyl radical to 1.60 Å, characteristic for an Mn=O bond, and fixing that distance in the optimization still led to an oxygen radical with spin 0.57, not an oxo. Since Mn(V)=O in the WU mechanism should be five-coordinated with an empty site trans to the oxo, the distance to the water trans to the oxyl was extended in steps of 0.3 Å. After extending the distance by 0.9 Å, the intended oxo ligand was still more like an oxyl radical with a spin of 0.43 even though the Mn–oxo distance was kept short at 1.60 Å. The energy went strongly uphill by +14.2 kcal/mol as the Mn–H_2_O distance was increased. One reason for that was the strong hydrogen bond between Asp61 and the water on Mn4, making the water hydroxide like. It should be recalled that Asp61 was removed in the WU mechanism.

**Figure 3:**
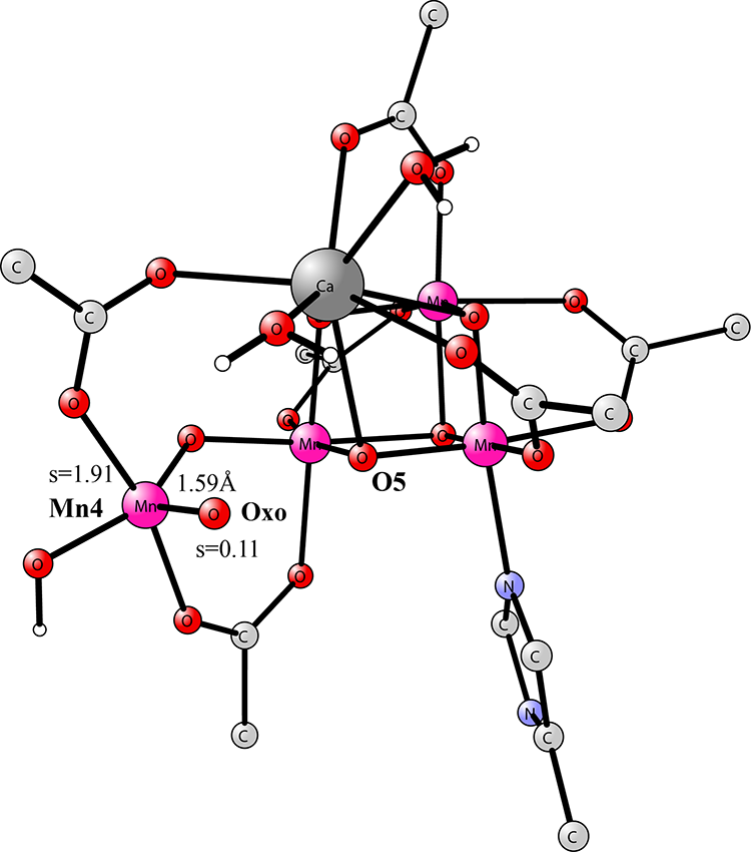
Optimized five-coordinated Mn(V)–oxo structure.

Instead, progress to reach a Mn(V)=O structure was reached when the water ligand on Mn4 was simply removed from the model. The oxygen radical was then transformed to an oxo ligand with an optimized Mn–O distance of 1.59 Å and a spin of 0.11, typical for Mn(V)=O. The spin on Mn4 is 1.91. The other ligands on Mn4 became bent somewhat toward the empty site. The structure is shown in Figure 3. When the water molecule was brought back into the model, placed among the waters around Mn4 but not on Mn4, the oxo character remained with a distance of 1.59 Å.

**Figure 4:**
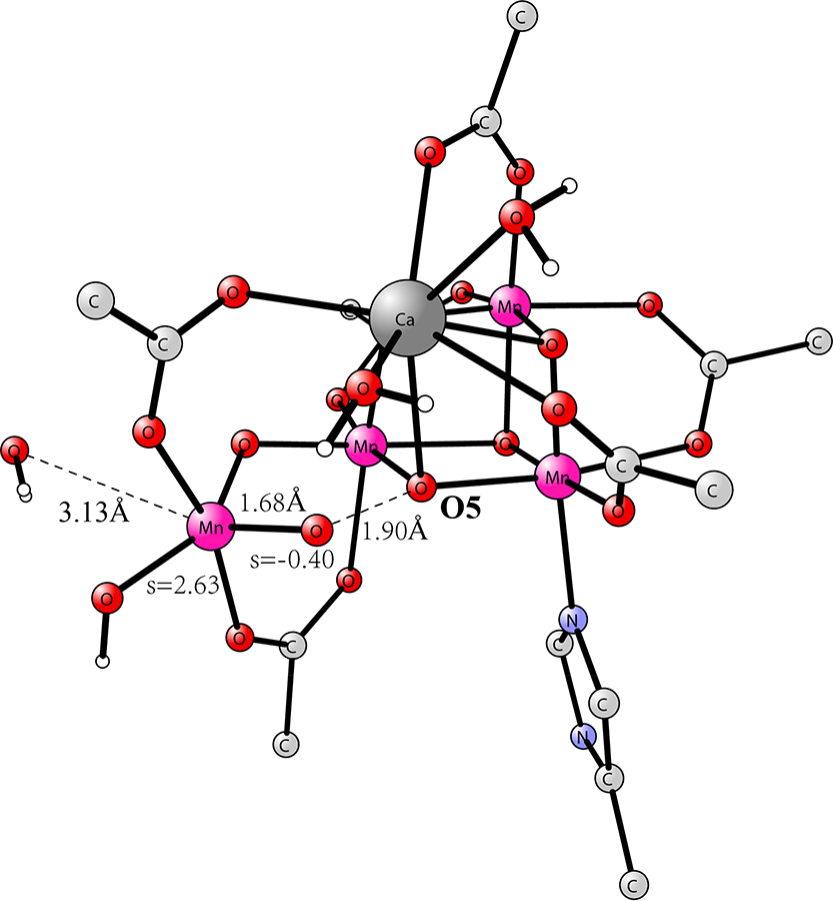
Approximate TS for O–O bond formation for the Mn(V)–oxo structure. It still has a five-coordinated Mn4, but a water is not far away. The barrier from the Mn(V)–oxo structure is +12.6 kcal/mol.

The most interesting result came when the O–O transition state region of the WU mechanism was approached, moving the oxo group in Mn(V)=O toward O5 in the closed cubane. The approximate TS has a O–O distance of 1.9 Å, see Figure 4. The calculated barrier for O–O bond formation is +12.6 kcal/mol in good agreement with the results in the Messinger and co-workers study. The spin on the oxo has now changed from +0.11 in the Mn(V)–oxo structure to −0.40, and the one on Mn4 from 1.91 to 2.63, indicating a large change from Mn(V)=O toward Mn(IV)–oxyl. Therefore, the TS is not of oxo–oxo, as claimed for the WU mechanism,^14^ but of oxyl–oxo type. An outside water molecule now has a rather short distance to Mn4 with a distance of 3.13 Å.

**Figure 5:**
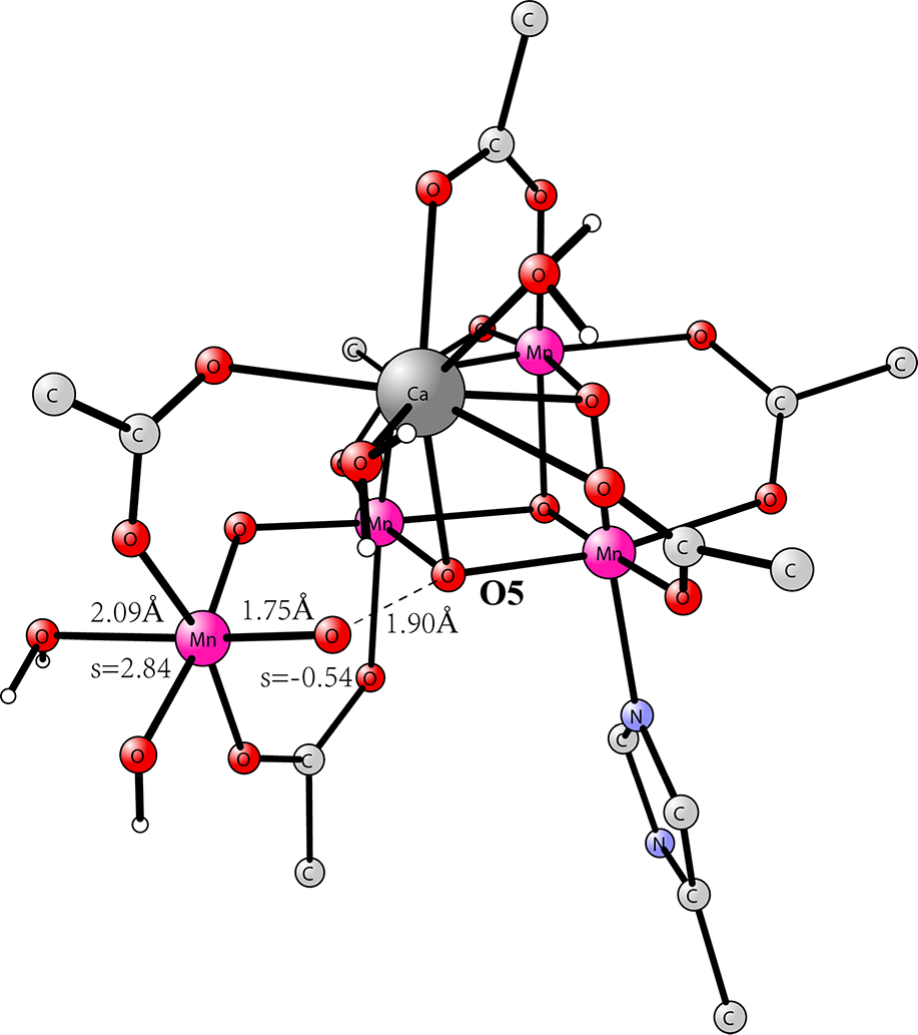
Approximate TS structure for the closed cubane when the water molecule has become bound to Mn4.

It turned out that the water molecule from the surrounding of Mn4 could be moved, without any barrier, toward the empty site of Mn4. The Mn4–H_2_O distance has at the end decreased to 2.09 Å, indicating a rather strong bond. As the water molecule approached, the spin on the oxyl radical became −0.54. The spin on Mn4 has increased to 2.84 from 1.91 for the Mn(V)–oxo structure. The energy goes sharply down by −11.1 kcal/mol when the water is moved closer to Mn4. The TS energy for the six-coordinated structure in Figure 5 is only 1.5 kcal/mol above the energy of the five-coordinated Mn(V)=oxo structure in Figure 3. The approximate TS structure in Figure 5 is very similar to the one found in our earlier study for mechanism D from 2015, but the water binding pattern around the OEC was different. Choosing another pattern, the energy decreases by −7.0 kcal/mol, but it would not be compatible with the other structures studied here. Considering the present results, the WU mechanism is not new, but instead similar to mechanism D, but with important differences. One difference is the starting point for the O–O bond formation which is the Mn(V)–oxo structure in Figure 3 for the WU mechanism, while it is an oxyl radical structure for mechanism D. However, that is not an improvement since the energy for the Mn(V)–oxo structure is 8.3 kcal/mol higher than the oxyl structure on the same potential surface. It is also +13.5 kcal/mol higher than the open cubane structure in Figure 2. It can be added that for the energetics for the movement of the water, it is important to have a balanced treatment from the distance of 3.13 to 2.09 Å. For that reason, the water at 3.13 Å has strong hydrogen bonds, both to Arg357 and to Asp61.

To complete the picture, calculations were finally done without Asp61 since that was the model used for the WU mechanism.^14^ The charge of the model is then +2 instead of +1. The reason for leaving Asp61 out, given by Pantazis et al., was that there were technical difficulties if Asp61 was included. No such difficulties were found in the present study. The modeling was absolutely normal with or without Asp61. Without Asp61, the six-coordinated Mn4(IV)–oxyl was still found to be better than Mn(V)=O, now by 4.0 kcal/mol. A few water binding patterns were tried without significant differences. With Asp61, the difference between the two structure was +8.3 kcal/mol as mentioned above.

## 4 Conclusions

The recently suggested WU mechanism for water oxidation in PSII by Messinger and co-workers^14^ has here been reinvestigated. It was suggested that the WU mechanism should be the preferred one in higher plants. The reason was a previous conclusion by Pantazis et al., who suggested a closed cubane structure for S_3_ based on an EPR analysis for the S = 6 structure for higher plants.^11−13^ However, the energy for the closed cubane structure in S_3_ with a five-coordinated Mn4 had previously been found to be much higher than the open cubane structure, both shown in Figure 1.^15^ The energy difference is here found to be +14.9 kcal/mol. With the accuracy typical for the methods used here of about 3 kcal/mol,^16^ the closed cubane structure must, therefore, be ruled out as a possible S_3_ state. Another recent EPR analysis has instead concluded that S_3_ has an open cubane structure.^24^ Other S = 6 structures than the one found by Pantazis et al. could, for example, be obtained by simply reversing the signs of the spins on some Mn(IV) centers and keep the open cubane. That should only marginally affect the energies and, therefore, not be important for the oxyl–oxo mechanism.

**Figure 6:**
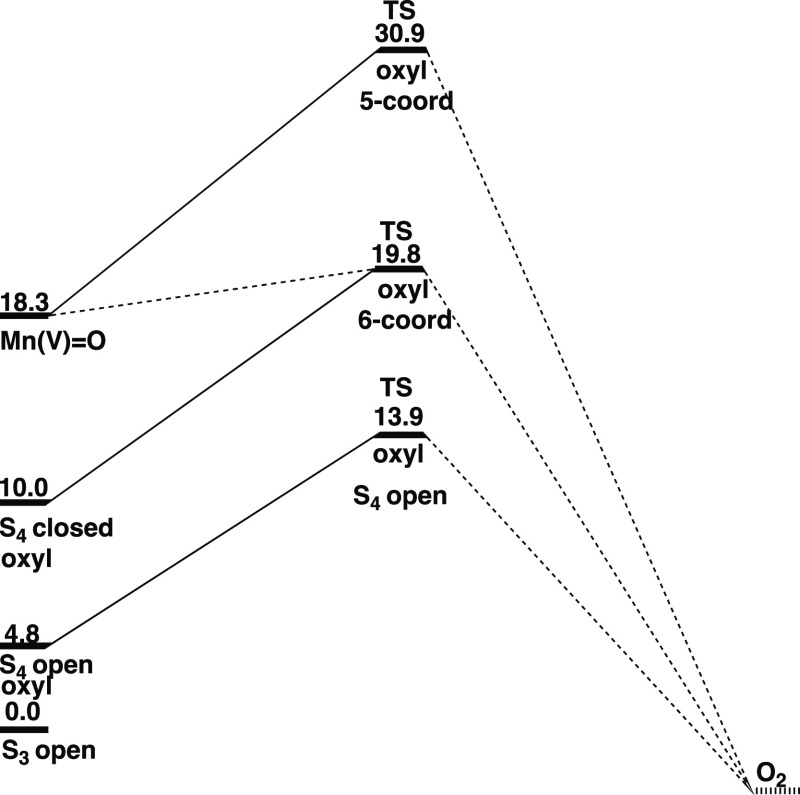
Energy diagram showing both the old mechanism, termed S_4_ open, and the WU mechanism termed Mn(V)=O with the full line.

In the WU mechanism, which was based on the conclusions by Pantazis et al., a closed cubane structure with a five-coordinated Mn4 was used as the ground state for S_4_. The optimization led to a Mn(V)=O for Mn4, see Figure 3. However, in the present study, the Mn(V)=O structure is found to be +16.2 kcal/mol higher in energy than the open cubane structure for S_4_. Starting with the Mn(V)=O structure and approaching a TS structure, here led to a similar TS as found in the WU mechanism with an approximate barrier of +12.6 kcal/mol with respect to Mn(V)=O.^14^ The wave function for the TS has undergone a drastic change from the one of Mn(V)=O, and is now much more Mn(IV)-oxyl like. Also, approaching outside water toward the empty site of Mn4 led to a large energy decrease for the TS energy by −11.1 kcal/mol. The six-coordinated Mn4 TS is now very similar to the one found for mechanism D in a previous study from 2015.^25^ That mechanism is in turn very similar to the open cubane mechanism. It involves the same Mn atoms in an antiferromagnetic coupling, and the O5 oxo is part of the O_2_ formed. The conclusion here is that the WU mechanism is just a modification of the previous mechanism D but with a ground state that is +8.3 kcal/mol higher and a TS that is +11.1 kcal/mol higher in energy. The main differences in the two modeling studies are, first, that a water molecule was allowed to bind to the empty site of Mn4 in the present study, but in the previous study of the WU mechanism, that possibility was not considered. Second, by removing a negative Asp61, as done for the WU mechanism, the charge of the OEC and its immediate surrounding increases by one plus-charge. That charge change should be present also in the earlier S states with far reaching consequences for the energetics. The energy diagrams for the old mechanism^9,25^ and the ones of the present study are shown in Figure 6.

## Supporting Information Available

Coordinates for all structures (PDF)
