# Supplementary material for: Is There a Different Mechanism for Water Oxidation in Higher Plants?
Source: J Phys Chem B. 2023 Jul 19;127(30):6643–7. doi: 10.1021/acs.jpcb.3c03029 (PMC10405216; doi:10.1021/acs.jpcb.3c03029)
Supplement: Supplementary file 1 — jp3c03029_si_001.pdf [file jp3c03029_si_001.pdf]

Supporting information:

Is There a Different Mechanism for Water Oxidation in Higher Plants ?

Yu-Tian Song, Xi-Chen Li*

College of Chemistry, Beijing Normal University, Beijing, China.
Email: li_xi_chen@hotmail.com

Per E. M. Siegbahn*

Department of Organic Chemistry, Arrhenius Laboratory, Stockholm University, SE-106 91, Stockholm, Sweden. Email:per.siegbahn@su.se

The open cubane structure for S_4 in **Figure 2.** # means fixed atom
Energies: E= -5850.480316, solv = -0.126649, disp = -0.405227
The spins on Mn: -2.97, 2.88, 2.93, 2.98 ; O188-spin -0.61

Mn1	3.0206729811	-1.4183020435	3.8592793666
Mn2	4.5998389217	-1.4895770765	8.6584320507
Mn3	5.3547737340	-1.5091719976	5.3331353455
Mn4	6.6101653984	-0.0202258703	7.3777054267
O5	3.6102537423	-1.1794885383	5.6015784944
O6	5.7945041973	0.2162749355	5.8037542397
O7	5.1094687609	0.2638949269	8.3681796272
O8	5.9400002742	-1.8096714841	7.3375767652
Ca9	3.4221391065	1.1475831737	6.7963326896
C10	0.6675460032#	1.9515099547#	1.7063470076#
H11	1.2251839950#	1.9058030158#	0.7825779962#
H12	-0.3243899933#	2.3686410157#	1.6143239994#
H13	0.4729496046	0.8952650788	1.9391249897
C14	1.4868828573	2.5223992172	2.8788598899
H15	0.8181183238	2.9005529520	3.6630144561
H16	2.0967833035	3.3853170354	2.5842090454
C17	2.4338900058	1.5667831898	3.6424985081
O18	3.0806694599	2.0243098331	4.6068273755
O19	2.4784364966	0.3406445857	3.2406501922
C20	1.0582600139#	1.1829969850#	13.4563139981#
H21	0.3702239974#	0.3601390023#	13.5824630009#
H22	0.7336469954#	2.1291270020#	13.8635789916#
H23	1.9409907446	0.9077120559	14.0533865056
C24	1.4750860164	1.2356374806	11.9851404791
H25	2.3153038395	1.9275666653	11.8431720164
H26	0.6553921604	1.5882999911	11.3486895537
C27	1.8898751054	-0.1805642399	11.5608422154
H28	1.0021971806	-0.8295763059	11.5233124513
H29	2.5672602794	-0.6204601545	12.2993885611
C30	2.5541513505	-0.2617404642	10.2044593316
O31	2.1820233243	0.4301337236	9.2476228842
O32	3.5345552593	-1.1314142487	10.1758200686
C33	2.7844500062#	-7.8608079881#	8.6257439953#
H34	2.4383166484	-8.8273984498	9.0128435927

H35	1.9660291498	-7.4237477518	8.0476747766
C36	4.0036564563	-8.1029471586	7.7335557228
O37	5.1329041558	-8.3110497809	8.2249686970
C38	3.1404651865	-6.9608361654	9.8456168789
H39	3.8368730995	-7.5096648551	10.4906930745
H40	2.2337389881	-6.7771599687	10.4289883389
C41	3.7530746250	-5.6432653524	9.4542419696
N42	4.8631634636	-5.6380372573	8.6264834846
H43	5.3413553969	-6.4983188024	8.3438132601
C44	3.4477877015	-4.3122102160	9.6381016705
H45	2.6635670489	-3.8571539394	10.2225177605
C46	5.1524518427	-4.3772171996	8.2842382634
H47	5.8680395165	-4.1024273955	7.5334376939
N48	4.3376993526	-3.5370537308	8.9061913697
N49	3.8038986041	-7.9556299484	6.4101162659
H50	2.8679093704	-7.6801345169	6.0959039762
C51	4.9172529926#	-7.8463449884#	5.4688660042#
H52	4.6502419362	-8.3899611013	4.5541944158
H53	5.7808223799	-8.3372300325	5.9196417393
C54	5.2594325144	-6.3669221447	5.1252506116
H55	6.1230484218	-6.3645912402	4.4471142320
H56	5.5935939546	-5.8726749963	6.0403772039
C57	4.0992084398	-5.5600009452	4.5031539562
H58	3.1411383320	-5.8096733767	4.9786287316
H59	3.9656006723	-5.8063496869	3.4428500428
C60	4.1808356645	-4.0373185571	4.5831784361
O61	3.2240280194	-3.4029464506	4.0355985138
O62	5.1461675250	-3.4907856831	5.2079150982
C63	9.1595120035#	0.1191579986#	11.4478230039#
H64	9.8466259681	-0.1516688253	10.6391046622
H65	9.7031143489	-0.0063325204	12.3934089898
C66	7.9321502934	-0.7946049643	11.4540802908
H67	7.2102171274	-0.4478676116	12.2001019770
H68	8.2173692229	-1.8109734400	11.7552017982
C69	7.1786383198	-0.8995247370	10.1293476832
O70	7.6587539796	-0.3054002760	9.1174141731
O71	6.1332555310	-1.6218489374	10.1258939914
C72	8.7681777834	1.5940847959	11.3279544017
O73	7.6357840884	2.0033226919	11.6046220331
N74	9.7759531931	2.4284504448	10.9701207334
H75	10.6311329743	2.0290854499	10.6072939334
C76	9.5677779932#	3.8588149977#	10.7651889942#
H77	10.5122491901	4.3808763148	10.9254552049
H78	8.8314136948	4.1990162012	11.4965316287
C79	9.1136881109	4.1421758789	9.3133015107
O80	9.9244960682	4.4253987150	8.4381738450
N81	7.7708343176	4.0269276876	9.1016031910
H82	7.1774588624	3.6588183463	9.8367015729
C83	7.1868535309	4.1233079286	7.7790725446
H84	6.4299841718	4.9123785075	7.7388951248
H85	8.0016989448	4.3759763179	7.0926247167
C86	6.5127006644	2.8215123250	7.3207002647
O87	5.3807613958	2.8486007642	6.8211698083
O88	7.2397148349	1.7547064284	7.5006867979
C89	11.5816619955#	-0.6170830073#	3.4808990000#
H90	12.4441750022#	-1.2180929968#	3.7284030001#
H91	11.8304269999#	0.3821370006#	3.1552080019#
H92	11.1186161033	-1.0964117840	2.6069777018
C93	10.5308318588	-0.4646122550	4.5882102752
H94	9.8985780694	0.3968322408	4.3397601203
H95	10.9889639789	-0.2257059769	5.5541219538
C96	9.6266564740	-1.6959650923	4.7117125214
H97	10.1529457286	-2.4935171318	5.2535656898
H98	9.3883359578	-2.1012662349	3.7241420364

C99	8.3026311550	-1.3953763206	5.3947217265
O100	7.2713094631	-1.8736088989	4.8292261351
O101	8.3077309080	-0.7024991695	6.4622465275
C102	10.8187249950#	4.2285689896#	-0.8150210023#
H103	11.5403640042#	3.4839220037#	-0.5131310009#
H104	11.2010929962#	5.2306260005#	-0.6882239927#
H105	10.6663006454	4.0975744187	-1.8933182097
C106	9.4834360418	4.0169169986	-0.0828898219
H107	9.0378216310	3.0826203110	-0.4567672639
H108	8.7803774478	4.8174127912	-0.3510411380
C109	9.6269900202	3.9517866854	1.4453980045
H110	10.0874413106	4.8799739843	1.8073815365
H111	10.3090572135	3.1365210622	1.7216837367
C112	8.3059162635	3.7750754679	2.2075783612
H113	7.6329943305	4.6169765037	1.9867924926
H114	8.5016528517	3.7765909740	3.2847739089
N115	7.6533758614	2.5045487224	1.8774082469
H116	7.7081218351	2.1866271540	0.9191344146
C117	6.8106293111	1.8168675691	2.6762304447
N118	6.4130175634	2.3293011415	3.8500194265
H119	5.9723350863	1.7226151582	4.5446495717
H120	6.3670483075	3.3243807444	4.0093591113
N121	6.3754670702	0.6157865594	2.3011083119
H122	6.9060263932	-0.0491441760	1.7287789366
H123	5.6717636538	0.1274195795	2.8700702757
C124	11.8423979984#	-5.3930899978#	7.2492549994#
H125	11.2522020008#	-5.9724610005#	6.5547079997#
H126	12.7964629996#	-5.8524490004#	7.4617420009#
H127	12.0219882689	-4.3946345315	6.8382763165
C128	11.1607723600	-5.3388275377	8.6316010623
H129	11.8464983631	-4.8669758668	9.3497751105
H130	11.0113845393	-6.3660903738	8.9908402335
C131	9.8560899677	-4.6149923405	8.6840425217
N132	9.0009456880	-4.7001709080	9.7789143896
C133	9.2520915038	-3.7368383807	7.8292522036
H134	9.5652970298	-3.3797820690	6.8662552866
C135	7.9332261797	-3.9103363117	9.5927422218
H136	7.1074810995	-3.7583004572	10.2701432567
N137	8.0769276588	-3.3134676957	8.4141043528
H138	7.3657175725	-2.6698507031	7.9924096448
C139	6.0989359749#	-4.2351879956#	-1.2221969849#
H140	5.3833630115#	-4.5684340076#	-1.9592870078#
H141	7.1094170010#	-4.1301810018#	-1.5886719978#
H142	5.8002554601	-3.2124515870	-0.9618126757
C143	5.9836490683	-5.0866471524	0.0591052616
H144	5.9690088252	-6.1578656928	-0.1638412102
H145	6.8407320693	-4.8783602601	0.7108050911
C146	4.7167374357	-4.6774093125	0.8157960625
O147	4.6810792379	-3.4805816496	1.2212021424
O148	3.7688756922	-5.5169220726	0.9676770080
C149	-1.8288759690#	-11.9867179948#	4.2485679971#
H150	-2.3586930107#	-12.7180949929#	3.6563010008#
H151	-2.3284060067#	-11.7832499942#	5.1842339952#
H152	-0.8512245157	-12.4154514795	4.5057536807
C153	-1.6507571803	-10.6549230928	3.4832946399
H154	-2.5525340927	-10.4588830474	2.8856967200
H155	-1.5871748295	-9.8332685233	4.2086182089
C156	-0.4114632645	-10.6140422757	2.5727835442
H157	0.4930137148	-10.5512634007	3.1954778820
H158	-0.3297888074	-11.5630333420	2.0255407770
C159	-0.4269590939	-9.4874603085	1.5232787826
H160	0.4670308209	-9.5517466140	0.8883163595
H161	-1.2836762839	-9.6552491792	0.8568479196
C162	-0.5615352158	-8.0454237627	2.0270244638

H163	-0.8378709911	-7.3993160035	1.1893322047
H164	-1.3371501505	-7.9424296262	2.7892774840
N165	0.7031257679	-7.4595029142	2.6022555608
H166	0.8055383772	-6.4768415364	2.2604618689
H167	0.7122822219	-7.4037316670	3.6451622387
H168	1.5723296797	-7.9300203593	2.2649874806
Cl169	0.8670815516	-6.5961910425	5.6562043185
O170	1.2718754904	-1.6132784733	4.3912047815
H171	0.3236088331	-2.1406745829	2.8461034090
O172	4.8867458294	-1.2550051606	3.6587939610
O173	7.1157006153	-2.0411065855	1.7392796021
H174	7.0444565528	-2.0967933682	2.7086570316
H175	6.2761606545	-2.4638473265	1.4440993411
O176	1.0994120251	0.3826467298	6.3618989171
H177	1.0395145619	-0.2690810108	5.6155606254
H178	0.7684327625	-0.0986752439	7.1374791547
O179	2.9099156876	2.9268247771	8.4639864987
H180	3.6951908873	3.1263915981	9.0139770175
H181	2.3192304917	2.4461609688	9.0722325529
O182	5.1732980348	2.4209465380	10.1542397050
H183	5.2386783717	1.5900860621	9.6368296220
H184	5.8028722016	2.3019905614	10.8893745418
O185	2.8390978936	-1.8012040692	1.9385678229
H186	3.4417808049	-2.5706952714	1.6775220408
H187	1.0136885125	-4.2789057741	6.2844841874
O188	3.1981074686	-1.3944618785	7.6988078647
O189	1.1872299961	-5.0064970932	1.3235490775
H190	2.1935032888	-5.0478561276	1.2830964338
H191	0.9128482393	-5.0305320087	0.3922766348
O192	0.3107775544	-2.4059681936	1.8976552325
H193	0.4567302820	-3.3770561468	1.8945346689
H194	1.8622409917	-2.0142487629	1.7488583848
O195	3.1304830564	-8.1252511677	1.3863364253
H196	3.4897114522	-7.2429746058	1.1134617938
H197	3.8962094227	-8.6702098419	1.6151723946
O198	1.0502889397	-3.3203732777	6.4911346603
H199	1.2016814732	-2.3449083978	5.0694827002
H200	1.8974247737	-3.1574717395	6.9386237549
H201	9.1497407938	-5.2791314338	10.5962219358

The closed cubane structure for S_4 in **Figure 2.**

Energies: E= -5850.466744, solv= -0.134509, disp= -0.398247
The spins on Mn: 2.92, -3.35, 2.84, 2.94 ; O188-spin -0.61

Mn1	2.61813829	-1.45835371	4.05788035
Mn2	4.70071409	-1.45145938	8.60514536
Mn3	5.26391361	-1.52088838	5.75920523
Mn4	6.68285922	0.10530455	7.50100666
O5	3.88803462	-1.36582244	6.98626648
O6	5.73724769	0.24853411	6.01153535
O7	5.17037598	0.32558268	8.49827093
O8	6.21394097	-1.75476245	7.52891811
Ca9	3.43226717	1.28108659	6.86841686
C10	0.66765300#	1.95117500#	1.70645900#
H11	1.22513700#	1.90593300#	0.78254000#
H12	-0.32435100#	2.36874600#	1.61430400#
H13	0.45707700#	0.89593300#	1.92231000#
C14	1.47496291	2.48635171	2.89716589
H15	0.79946469	2.87964299	3.66861718
H16	2.13991045	3.31702611	2.63417859
C17	2.32658203	1.45184068	3.65352701
O18	3.11068165	1.82692832	4.54631950
O19	2.12255566	0.21969598	3.31576148

C20	1.05826500#	1.18289500#	13.45621700#
H21	0.37021000#	0.36015900#	13.58249700#
H22	0.73366100#	2.12914400#	13.86359100#
H23	1.94740800#	0.90731000#	14.04281700#
C24	1.46320955	1.26611905	11.96725027
H25	2.25383305	2.01510812	11.83155909
H26	0.61302860	1.58888719	11.35428760
C27	1.96817884	-0.10102667	11.47131545
H28	1.15525431	-0.84167910	11.51898049
H29	2.75182374	-0.48435647	12.13528273
C30	2.52406502	-0.15546374	10.05153811
O31	2.25138079	0.68592206	9.18500657
O32	3.29497202	-1.19054098	9.83746962
C33	2.78434400#	-7.86067400#	8.62583600#
H34	2.47664000#	-8.81843200#	9.06309200#
H35	1.95396100#	-7.50500600#	8.00848200#
C36	4.02219439	-8.08204258	7.74727401
O37	5.16183083	-8.18304212	8.25770196
C38	3.07673150	-6.86576901	9.79190958
H39	3.73441967	-7.36730341	10.51189356
H40	2.13803026	-6.65125291	10.31108585
C41	3.71973232	-5.56625686	9.37860607
N42	4.93039765	-5.59473390	8.70941364
H43	5.39313670	-6.48544909	8.47415295
C44	3.40126042	-4.22764593	9.47183114
H45	2.55372393	-3.74601673	9.93302972
C46	5.27338216	-4.34938547	8.36917856
H47	6.09394084	-4.10978778	7.72174192
N48	4.38671793	-3.48118944	8.83915848
N49	3.82718083	-8.03169880	6.42273160
H50	2.86315595	-7.98460850	6.05281333
C51	4.91755300#	-7.84623300#	5.46887900#
H52	4.63564700#	-8.34292700#	4.53514900#
H53	5.82073900#	-8.31857400#	5.85830100#
C54	5.18940792	-6.33269878	5.20948808
H55	6.01685488	-6.24860218	4.49269970
H56	5.54944697	-5.89245162	6.14225610
C57	3.97292145	-5.52823847	4.69845854
H58	3.04070716	-5.87205018	5.16587083
H59	3.82518210	-5.68568023	3.62240574
C60	4.00046619	-4.00828520	4.88839897
O61	2.97257122	-3.38799871	4.46773952
O62	5.00660255	-3.46450978	5.45093867
C63	9.15949800#	0.11914400#	11.44782200#
H64	9.77292700#	-0.15994400#	10.58639500#
H65	9.77971500#	-0.01134200#	12.34551700#
C66	7.92501049	-0.77020327	11.54611845
H67	7.24498299	-0.40139097	12.32003892
H68	8.20407398	-1.79090998	11.83615680
C69	7.14582216	-0.83964158	10.24069530
O70	7.66225575	-0.24008033	9.24618959
O71	6.07694307	-1.52575520	10.23376822
C72	8.78176377	1.59786212	11.35864033
O73	7.67206642	2.01296243	11.70764562
N74	9.77170643	2.42519979	10.94322045
H75	10.60481139	2.02215182	10.53584338
C76	9.56769600#	3.85878600#	10.76514600#
H77	10.51000700#	4.37669200#	10.95467100#
H78	8.82129800#	4.17845700#	11.49446300#
C79	9.12990182	4.16839188	9.31911136
O80	9.95546500	4.38087563	8.43923472
N81	7.77978568	4.16099598	9.11589268
H82	7.16814455	3.80844922	9.84390264
C83	7.20488039	4.25064264	7.79078896

H84	6.44242690	5.03352083	7.74272481
H85	8.02216362	4.50500235	7.10741338
C86	6.55137074	2.94167970	7.33094036
O87	5.46096041	2.95190977	6.75087566
O88	7.25486815	1.87742701	7.60470036
C89	11.58174700#	-0.61712300#	3.48092100#
H90	12.44418600#	-1.21807000#	3.72839500#
H91	11.83037600#	0.38213900#	3.15519200#
H92	11.11981100#	-1.09652600#	2.60655500#
C93	10.53275533	-0.48116159	4.61025993
H94	10.04413879	0.49561846	4.51632040
H95	11.00892553	-0.47256137	5.59703025
C96	9.43749287	-1.55528560	4.54871694
H97	9.81920497	-2.53221760	4.88500800
H98	9.11477287	-1.71780526	3.51508199
C99	8.18129489	-1.24784558	5.35835311
O100	7.10134722	-1.76382013	4.91747128
O101	8.29262589	-0.53647529	6.40673995
C102	10.81873400#	4.22853500#	-0.81501400#
H103	11.54037000#	3.48392900#	-0.51313100#
H104	11.20108300#	5.23063200#	-0.68823000#
H105	10.67111100#	4.10254400#	-1.89439600#
C106	9.47467837	3.97570420	-0.09556171
H107	8.99133581	3.10757811	-0.56814991
H108	8.79728554	4.82476350	-0.25851940
C109	9.62193299	3.72142282	1.41432032
H110	10.14453578	4.57308125	1.86903011
H111	10.25791142	2.84281440	1.58853502
C112	8.31073073	3.55222271	2.20101292
H113	7.64063859	4.39714006	1.98036449
H114	8.53956507	3.57953360	3.27129152
N115	7.61871115	2.27811397	1.94764797
H116	7.65975152	1.88706268	1.01576999
C117	6.70697928	1.73490977	2.78862284
N118	6.35939574	2.39771371	3.90297911
H119	5.82512485	1.92102751	4.62539922
H120	6.40054318	3.40372939	3.95712905
N121	6.16315475	0.54463194	2.54059538
H122	6.63056860	-0.22905450	2.03762896
H123	5.42780316	0.20047460	3.16570866
C124	11.84244700#	-5.39311800#	7.24929500#
H125	11.25217800#	-5.97244000#	6.55469900#
H126	12.79646100#	-5.85244700#	7.46172300#
H127	12.01938800#	-4.39035500#	6.84896500#
C128	11.19718276	-5.36016400	8.64933189
H129	11.92380588	-4.94728182	9.36366755
H130	11.01052480	-6.39227104	8.97507076
C131	9.93490051	-4.57974342	8.76283503
N132	9.07585667	-4.70661199	9.84919753
C133	9.37674667	-3.61192602	7.97502160
H134	9.71058290	-3.18796077	7.04349128
C135	8.04412658	-3.85875103	9.72090074
H136	7.21061304	-3.73978105	10.39563232
N137	8.21993227	-3.18541415	8.58960344
H138	7.50978328	-2.52181807	8.17952518
C139	6.09855600#	-4.23516900#	-1.22214400#
H140	5.38344900#	-4.56843900#	-1.95933800#
H141	7.10947000#	-4.13021100#	-1.58863200#
H142	5.79539700#	-3.21949900#	-0.93673100#
C143	6.07644623	-5.14504098	0.02945256
H144	6.06886218	-6.20113181	-0.25749178
H145	6.97940979	-4.95638675	0.62182187
C146	4.87364433	-4.82649062	0.91629643
O147	4.90794041	-3.71953021	1.52962075

O148	3.90669730	-5.64931809	0.97912674
C149	-1.82881200#	-11.98640500#	4.24869900#
H150	-2.35863300#	-12.71815900#	3.65628000#
H151	-2.32848200#	-11.78331600#	5.18422000#
H152	-0.85738200#	-12.42670300#	4.51265600#
C153	-1.62614981	-10.68052181	3.45441270
H154	-2.57666452	-10.40528510	2.97337430
H155	-1.37674512	-9.86587416	4.14521199
C156	-0.51850487	-10.79460113	2.39077386
H157	0.45916740	-10.71678867	2.88652026
H158	-0.54831488	-11.79570980	1.93856891
C159	-0.61887772	-9.78456725	1.23366374
H160	0.18130556	-9.97246947	0.50384694
H161	-1.56166824	-9.96296000	0.69871907
C162	-0.61090416	-8.29553258	1.58751243
H163	-0.86015507	-7.70945472	0.69771029
H164	-1.34250212	-8.05670842	2.36376420
N165	0.71388508	-7.78873107	2.07614247
H166	0.78690204	-6.76730328	1.89935871
H167	0.83974604	-7.93238838	3.11856872
H168	1.53022118	-8.18268032	1.55655056
Cl169	0.94027802	-7.85005838	5.14275054
O170	0.80424250	-1.95433164	3.76676688
H171	0.44226027	-2.22389446	1.98944043
O172	4.41956015	-1.09410621	4.29195430
O173	7.05233205	-2.02167151	1.99598997
H174	6.98306005	-2.12656315	2.96333360
H175	6.32529611	-2.60369941	1.66142374
O176	0.97857707	1.48707417	6.82635123
H177	0.44823408	1.02145919	6.12489238
H178	0.69212834	1.10290560	7.67025870
O179	3.16690010	3.16899555	8.51507548
H180	3.93647111	3.27686834	9.11129718
H181	2.48434025	2.78779687	9.09419017
O182	5.29358541	2.51786898	10.24400456
H183	5.32594039	1.67454625	9.74353359
H184	5.93604258	2.39722944	10.96942901
O185	3.08802092	-1.88645610	2.12191479
H186	3.69475222	-2.66940907	1.98465533
H187	-0.06894326	-0.57266261	4.47988771
O188	2.15513976	-0.98918132	5.66816291
O189	1.29181831	-5.19808031	1.00210992
H190	2.28812387	-5.23222821	1.12232327
H191	1.17616638	-5.35437406	0.05003804
O192	0.74103437	-2.44110854	1.07679611
H193	0.81183330	-3.42213332	1.08007329
H194	2.24529627	-2.05059432	1.58537428
O195	3.04535467	-8.21938036	0.55020836
H196	3.48607229	-7.33535430	0.60882356
H197	3.72348776	-8.86022851	0.80923146
O198	-0.50845602	0.19375022	4.92944229
H199	-1.32775615	-0.16597447	5.30145895
H200	0.68230002	-2.77632769	4.27505031
H201	9.19808244	-5.35376957	10.61831342

The Mn(V)=O structure in **Figure 3.**
Energies: E= -5850.462433, solv= -0.133520, disp= -0.390375
The spins on Mn: 1.91, -2.97, 2.94, 2.95 ; O188-spin -0.11

Mn1	2.5773145883	-1.5591430037	4.0487329619
Mn2	4.6729797662	-1.4135984415	8.5806191479
Mn3	5.1999396203	-1.5183531787	5.7684180003
Mn4	6.6861505153	0.1077244316	7.4438968903

O5	3.8346248068	-1.3000834578	6.9913726706
O6	5.7094413184	0.2378465953	5.9701770359
O7	5.1921181002	0.3569223998	8.4709656403
O8	6.1650254597	-1.7510532120	7.4665735549
Ca9	3.5539900058	1.4101200584	6.8555817954
C10	0.6676530774#	1.9511748029#	1.7064590577#
H11	1.2251369633#	1.9059330674#	0.7825399745#
H12	-0.3243509754#	2.3687460602#	1.6143040092#
H13	0.4315950307	0.9006017403	1.9167862635
C14	1.4680208288	2.4557257292	2.9114910825
H15	0.7861267267	2.8269424733	3.6889039616
H16	2.1336593968	3.2942484844	2.6773972015
C17	2.3085969453	1.3944636700	3.6397927826
O18	3.1450302612	1.7696462242	4.5025024495
O19	2.0759385050	0.1748198572	3.3197785165
C20	1.0582650212#	1.1828949892#	13.4562169662#
H21	0.3702100014#	0.3601589984#	13.5824969981#
H22	0.7336609964#	2.1291440000#	13.8635909970#
H23	1.9529847633	0.9072500407	14.0337408927
C24	1.4507037771	1.2866491302	11.9653023921
H25	2.2474522930	2.0308516988	11.8382139260
H26	0.5996827759	1.6314602346	11.3657142661
C27	1.9362571149	-0.0752834868	11.4382260694
H28	1.1053505467	-0.7970289586	11.4393852933
H29	2.6926302682	-0.4989302938	12.1092198505
C30	2.5296923812	-0.0951003718	10.0330109854
O31	2.2959030284	0.7727884772	9.1812991621
O32	3.2945456896	-1.1379206829	9.8188424663
C33	2.7843440268#	-7.8606739870#	8.6258359690#
H34	2.5142258451	-8.8169195054	9.0899144850
H35	1.9449899599	-7.5539436706	7.9954401323
C36	4.0387991570	-8.0647009557	7.7622080620
O37	5.1779184715	-8.1517169714	8.2794512849
C38	3.0161878745	-6.8324457382	9.7782130293
H39	3.6546966851	-7.3034896479	10.5356797027
H40	2.0546911727	-6.6257514037	10.2570452440
C41	3.6534852157	-5.5304013068	9.3637300501
N42	4.8829469614	-5.5625820645	8.7288477397
H43	5.3571413236	-6.4537418024	8.5145138977
C44	3.3284227725	-4.1918225406	9.4315969185
H45	2.4659260120	-3.7090157891	9.8621297340
C46	5.2290058603	-4.3214537800	8.3793502648
H47	6.0619671848	-4.0902979332	7.7462538144
N48	4.3283352893	-3.4488857566	8.8145980260
N49	3.8414041429	-8.0251817824	6.4381111934
H50	2.8771501733	-7.9415646186	6.0812335567
C51	4.9175529548#	-7.8462329289#	5.4688790058#
H52	4.6106699304	-8.3354611599	4.5384550743
H53	5.8139007046	-8.3463501519	5.8423151922
C54	5.2220571030	-6.3420158416	5.2172573856
H55	6.0769073287	-6.2696509851	4.5317195150
H56	5.5490089236	-5.9018558935	6.1623846158
C57	4.0274778232	-5.5472410145	4.6565857961
H58	3.0795178896	-5.8938732127	5.0902278911
H59	3.9194975222	-5.7165440240	3.5778506061
C60	4.0176224930	-4.0343883425	4.8353911353
O61	3.0046034251	-3.4540526124	4.3404416349
O62	4.9669266339	-3.4537447409	5.4661685146
C63	9.1594979900#	0.1191439957#	11.4478219838#
H64	9.8020134741	-0.1541104360	10.6052642353
H65	9.7449641237	-0.0245595388	12.3659576922
C66	7.9172190608	-0.7673032718	11.5027449142
H67	7.2230675794	-0.4021386901	12.2656582377
H68	8.1863188747	-1.7906897494	11.7935758853

C69	7.1537160637	-0.8351072084	10.1855269034
O70	7.6830113617	-0.2507153464	9.1879480971
O71	6.0758986982	-1.5120922285	10.1772516047
C72	8.7868812032	1.6005643741	11.3678888011
O73	7.6787093327	2.0165861468	11.7228177721
N74	9.7787591895	2.4279045760	10.9571309694
H75	10.6101212234	2.0262340003	10.5440337823
C76	9.5676959746#	3.8587859948#	10.7651459859#
H77	10.4958746808	4.3899145436	10.9852014406
H78	8.7901996996	4.1762868834	11.4620097474
C79	9.1985481774	4.1456232242	9.2933221404
O80	10.0692993538	4.3167082051	8.4474789501
N81	7.8603096158	4.1616293547	9.0248371215
H82	7.2019202646	3.8438254576	9.7290523897
C83	7.3563228866	4.2341964673	7.6670890185
H84	6.6542305136	5.0646237549	7.5495818410
H85	8.2203160467	4.3980624438	7.0151753769
C86	6.6275506336	2.9508354188	7.2502478372
O87	5.5101110803	2.9971479231	6.7312401784
O88	7.3020202624	1.8604968734	7.5154684303
C89	11.5817469802#	-0.6171230206#	3.4809209786#
H90	12.4441860066#	-1.2180699877#	3.7283950072#
H91	11.8303760055#	0.3821390026#	3.1551920119#
H92	11.1343138716	-1.0953901868	2.5979618265
C93	10.5117061211	-0.5060264697	4.5985313069
H94	10.0612897370	0.4920890613	4.5551780078
H95	10.9674821553	-0.5731020827	5.5926950606
C96	9.3755818719	-1.5345269364	4.4590306334
H97	9.7184531356	-2.5461839949	4.7283013410
H98	9.0513070316	-1.6206725303	3.4165117796
C99	8.1254754472	-1.2464925775	5.2886492604
O100	7.0357952658	-1.7603611401	4.8668804066
O101	8.2593969283	-0.5480796001	6.3472830297
C102	10.8187339212#	4.2285348982#	-0.8150140947#
H103	11.5403700435#	3.4839290461#	-0.5131309898#
H104	11.2010829777#	5.2306319946#	-0.6882298908#
H105	10.7022217169	4.1068542719	-1.8998464378
C106	9.4452196320	3.9762654076	-0.1557705604
H107	8.9983096907	3.0949771317	-0.6392244392
H108	8.7702226690	4.8122042145	-0.3839215056
C109	9.4732805077	3.7586754297	1.3686642433
H110	9.9042737989	4.6421234767	1.8558484551
H111	10.1334969238	2.9175322822	1.6220909617
C112	8.0924683538	3.5234233845	2.0103983342
H113	7.4114685096	4.3247099669	1.6846694306
H114	8.1785391375	3.5807765440	3.1020773596
N115	7.5313073560	2.2090630405	1.6621123592
H116	7.9269201798	1.7323232081	0.8628082699
C117	6.3294535000	1.7311454867	2.0748659395
N118	5.5263391698	2.5001770300	2.8270681328
H119	4.7755030765	2.0970901144	3.3910887733
H120	5.7121570826	3.4837689509	2.9488919996
N121	5.9576829825	0.5067981061	1.7206339578
H122	6.6178138511	-0.2757141515	1.5829389374
H123	4.9797766376	0.1850928696	1.7491065537
C124	11.8424469947#	-5.3931179903#	7.2492949982#
H125	11.2521780052#	-5.9724400038#	6.5546989989#
H126	12.7964609976#	-5.8524470035#	7.4617230027#
H127	12.0234197745	-4.3933758748	6.8427010677
C128	11.1878983170	-5.3482491953	8.6407365056
H129	11.9045145682	-4.9259614588	9.3596475664
H130	10.9957912190	-6.3760148776	8.9769963596
C131	9.9255127007	-4.5658255642	8.7196461367
N132	9.0634899530	-4.6544167627	9.8064159347

C133	9.3700416414	-3.6304477600	7.8918136979
H134	9.7064030532	-3.2429490639	6.9455201392
C135	8.0323754853	-3.8139424539	9.6424874995
H136	7.1967598401	-3.6692874223	10.3092989966
N137	8.2112957397	-3.1825315253	8.4877685580
H138	7.5068319868	-2.5268009550	8.0680052716
C139	6.0985558825#	-4.2351690592#	-1.2221438086#
H140	5.3834490702#	-4.5684390164#	-1.9593380606#
H141	7.1094699762#	-4.1302109795#	-1.5886320598#
H142	5.7981139520	-3.2153406374	-0.9486609771
C143	6.0241518292	-5.1065436289	0.0516126194
H144	5.9762132389	-6.1714779433	-0.1951108728
H145	6.9226616035	-4.9375916102	0.6577326711
C146	4.8166068728	-4.7006174907	0.9030523649
O147	4.8296650852	-3.5459067866	1.4170723777
O148	3.8546513300	-5.5294816288	1.0384130923
C149	-1.8288116619#	-11.9864050020#	4.2486990784#
H150	-2.3586331189#	-12.7181588642#	3.6562799385#
H151	-2.3284821105#	-11.7833160073#	5.1842199426#
H152	-0.8469814133	-12.4066642524	4.5026101179
C153	-1.6643691258	-10.6473937386	3.4824466694
H154	-2.5954260204	-10.4359192883	2.9363328611
H155	-1.5433336652	-9.8331311143	4.2077410484
C156	-0.4749307831	-10.6160037802	2.5026590911
H157	0.4565336553	-10.5080500666	3.0754274057
H158	-0.4035349062	-11.5847661698	1.9886529203
C159	-0.5600937975	-9.5414710942	1.3977389399
H160	0.2591567672	-9.6868379532	0.6796802830
H161	-1.4854499378	-9.7111801794	0.8304779584
C162	-0.5812749280	-8.0611494272	1.8066972078
H163	-0.8957015105	-7.4596635627	0.9486973217
H164	-1.2774984147	-7.8684239582	2.6273110230
N165	0.7526683773	-7.5288331719	2.2439131105
H166	0.8107494864	-6.4940687978	2.1423397521
H167	0.9115077018	-7.7106934454	3.2741467149
H168	1.5512463347	-7.9015209298	1.6782721498
Cl169	0.9512063145	-7.5703498900	5.2738703761
O170	1.7170201168	-2.1371307759	2.6119873366
H171	2.8396170575	-1.0602927811	1.3388088524
O172	4.2319288953	-1.1039612790	4.3033440311
O173	7.0869006069	-2.0240917765	1.9358980840
H174	6.9090142828	-1.9829962485	2.8943898460
H175	6.3489861236	-2.5964768180	1.6040427597
O176	1.1133345319	1.5645207371	6.8321204239
H177	0.4421450739	1.2667989130	6.1725172033
H178	0.8022836241	1.2439783592	7.6939178475
O179	3.2435217329	3.2609841138	8.5200067983
H180	4.0127028604	3.3496207183	9.1215444089
H181	2.5529873872	2.8826753934	9.0915001721
O182	5.3757790901	2.5683467022	10.1973080910
H183	5.3922083438	1.7180884651	9.7076776262
H184	6.0010618976	2.4396897033	10.9371727324
H187	-0.8458235043	-0.0533552739	4.2410357007
O188	1.7000427050	-1.4177566234	5.3653693616
O189	1.4327579401	-4.7293608662	1.6961914218
H190	2.4047959442	-4.9575664291	1.5244985177
H191	1.1063282892	-4.4653611186	0.8200353486
O192	3.7251453381	-0.9825382105	0.9426258688
H193	4.0822206928	-1.8929364644	1.0315202159
O195	3.0102280216	-8.0877928458	0.6525997105
H196	3.4344541278	-7.1927252753	0.6625771473
H197	3.6858388058	-8.6910888782	0.9957136994
O198	-0.5751852576	0.2448559575	5.1361850334
H199	-0.0002349601	-0.4900359900	5.4193229768

H200	1.6973573444	-3.1311477506	2.5264076534
H201	9.1818578470	-5.2731683338	10.5992954271
O202	-0.9629121958	-1.0431821270	2.7026796835
H203	-0.0763300448	-1.4424300216	2.5895807845
H204	-1.4761170429	-1.2906431815	1.9211517416

The approximate O-O TS structure in **Figure 4.**

Energies: E= -5850.441877, solv= -0.132397, disp= -0.391920
The spins on Mn: 2.63, -3.28, 2.90, 2.94 ; O188-spin -0.40

Mn1	2.6891618883	-1.5317795808	4.0826541704
Mn2	4.6189470523	-1.4041294891	8.5448738999
Mn3	5.2901562087	-1.4772947753	5.6802056790
Mn4	6.6743640877	0.1084490425	7.4645786950
O5	3.7935140074	-1.2809225723	6.8162661735
O6	5.7599539734	0.2617836388	5.9463019250
O7	5.1479633530	0.3616137870	8.4268327749
O8	6.1322822786	-1.7342807116	7.4603074833
Ca9	3.5680432664	1.4368877029	6.7840733176
C10	0.6676530850#	1.9511747382#	1.7064590734#
H11	1.2251369541#	1.9059330877#	0.7825399678#
H12	-0.3243509662#	2.3687460827#	1.6143040125#
H13	0.4490605870	0.8985974402	1.9336927524
C14	1.4813213504	2.4753506565	2.8981182916
H15	0.8124503568	2.8649028876	3.6786219928
H16	2.1416446462	3.3090097100	2.6358282110
C17	2.3422439117	1.4328077310	3.6349784914
O18	3.2411457916	1.8354277204	4.4177400475
O19	2.0578952133	0.2022115238	3.4239710009
C20	1.0582650381#	1.1828949729#	13.4562169488#
H21	0.3702099988#	0.3601590008#	13.5824969995#
H22	0.7336609935#	2.1291440020#	13.8635909901#
H23	1.9561807501	0.9070566874	14.0286822043
C24	1.4385946483	1.3004507211	11.9645398696
H25	2.2237872559	2.0565679601	11.8369578501
H26	0.5778590877	1.6383960401	11.3749161663
C27	1.9370173801	-0.0479109975	11.4238887061
H28	1.1237865842	-0.7889767212	11.4415624781
H29	2.7182592559	-0.4585800057	12.0752429952
C30	2.5008597436	-0.0517115844	10.0091280275
O31	2.3061786574	0.8528094615	9.1888181307
O32	3.2021326913	-1.1271413596	9.7436681641
C33	2.7843440417#	-7.8606739818#	8.6258359532#
H34	2.5425182649	-8.8177561879	9.1030128716
H35	1.9266329337	-7.5781673943	8.0080562951
C36	4.0260699928	-8.0443274635	7.7495142144
O37	5.1694351417	-8.1165419509	8.2586529268
C38	3.0230253154	-6.8171176623	9.7639917633
H39	3.6788530642	-7.2745332434	10.5147081342
H40	2.0671302481	-6.6156539754	10.2560441414
C41	3.6392533973	-5.5128632045	9.3282382065
N42	4.8630757416	-5.5321728177	8.6826674407
H43	5.3458909684	-6.4194978696	8.4713280736
C44	3.2937797303	-4.1792574071	9.3882820193
H45	2.4294304996	-3.7050651120	9.8255027109
C46	5.1896396641	-4.2890808310	8.3237162230
H47	6.0164280890	-4.0467572494	7.6863491407
N48	4.2778964689	-3.4269793980	8.7585856939
N49	3.8267727925	-7.9963128300	6.4263039181
H50	2.8565173351	-7.9817029565	6.0650996025

C51	4.9175529267#	-7.8462329002#	5.4688790211#
H52	4.6159493568	-8.3432920557	4.5407589427
H53	5.8010162061	-8.3539910765	5.8619441363
C54	5.2466773432	-6.3523267470	5.2005882579
H55	6.0838903578	-6.3006486888	4.4921925231
H56	5.6060785255	-5.9102962567	6.1331591834
C57	4.0527728628	-5.5428203025	4.6638827658
H58	3.1165619125	-5.8571418545	5.1454267502
H59	3.9033578410	-5.7298974445	3.5932702231
C60	4.0914694365	-4.0244840111	4.8153757455
O61	3.0812926312	-3.4268679041	4.3237135075
O62	5.0570672560	-3.4553296823	5.4143344044
C63	9.1594979925#	0.1191439908#	11.4478219802#
H64	9.7975109520	-0.1545140750	10.6016027667
H65	9.7504608298	-0.0229469178	12.3627052155
C66	7.9185097286	-0.7701171110	11.5109382806
H67	7.2347276165	-0.4141098845	12.2874180045
H68	8.1959795359	-1.7949851183	11.7886814613
C69	7.1319945184	-0.8337564727	10.2054908103
O70	7.6493939088	-0.2412854393	9.2028747092
O71	6.0598130722	-1.5080823430	10.2001067774
C72	8.7843443171	1.5985100215	11.3659077249
O73	7.6718274545	2.0085386126	11.7117660683
N74	9.7770885552	2.4286439142	10.9626651446
H75	10.6114935958	2.0273969345	10.5555570072
C76	9.5676959642#	3.8587859926#	10.7651459795#
H77	10.4931880941	4.3907256844	10.9947732562
H78	8.7826672902	4.1772750856	11.4532926519
C79	9.2149484118	4.1393470017	9.2910414451
O80	10.0963014049	4.2841390193	8.4521710342
N81	7.8797985398	4.1779268622	9.0122296256
H82	7.2126039436	3.8699446821	9.7121784749
C83	7.3871695074	4.2342654897	7.6506172574
H84	6.6954363405	5.0706047675	7.5142097672
H85	8.2582887128	4.3762651798	7.0029354630
C86	6.6488271594	2.9521085000	7.2508092439
O87	5.5375910656	3.0024865263	6.7199889420
O88	7.3030854174	1.8592976276	7.5422483172
C89	11.5817469571#	-0.6171230439#	3.4809209594#
H90	12.4441860130#	-1.2180699761#	3.7283950134#
H91	11.8303760084#	0.3821390062#	3.1551920252#
H92	11.1250169030	-1.0961172485	2.6035013804
C93	10.5299989881	-0.4901578060	4.6062109867
H94	10.0492641510	0.4919102580	4.5288586448
H95	10.9996152230	-0.5057979672	5.5959048704
C96	9.4289813627	-1.5555436651	4.5137576786
H97	9.8013446333	-2.5422465674	4.8314132022
H98	9.1083954275	-1.6953895841	3.4762757690
C99	8.1771574905	-1.2517985418	5.3249602713
O100	7.0901606969	-1.7387637334	4.8612861899
O101	8.2880377034	-0.5777033554	6.3943331963
C102	10.8187339023#	4.2285348649#	-0.8150141318#
H103	11.5403700567#	3.4839290622#	-0.5131309816#
H104	11.2010829682#	5.2306319941#	-0.6882298577#
H105	10.7008161674	4.1073983317	-1.8997902558
C106	9.4527183246	3.9722624975	-0.1477292964
H107	8.9911081601	3.1056396301	-0.6430690834
H108	8.7799078201	4.8181723477	-0.3428069024
C109	9.5124856530	3.7165643119	1.3681984064
H110	9.9573755656	4.5852602423	1.8690695854
H111	10.1750002742	2.8667129501	1.5851971369
C112	8.1453314636	3.4713965602	2.0288117854
H113	7.4672246948	4.2884351682	1.7394160683
H114	8.2529559083	3.4956160982	3.1203089835

N115	7.5613538299	2.1771489215	1.6483712880
H116	7.9848604230	1.6764039865	0.8789344051
C117	6.3373669896	1.7358510386	2.0296629353
N118	5.5361156620	2.5380158990	2.7480985254
H119	4.7911196181	2.1514123041	3.3333707067
H120	5.8019696721	3.4921363076	2.9360679628
N121	5.9302365021	0.5254885490	1.6594157613
H122	6.5733436294	-0.2821027511	1.5695905354
H123	4.9534617070	0.2257608144	1.7610911846
C124	11.8424469949#	-5.3931179913#	7.2492950009#
H125	11.2521780062#	-5.9724400035#	6.5546989977#
H126	12.7964609980#	-5.8524470033#	7.4617230016#
H127	12.0238630120	-4.3946132829	6.8391934438
C128	11.1833320918	-5.3407836290	8.6415678608
H129	11.8985837514	-4.9099279782	9.3566540886
H130	10.9992002618	-6.3680163847	8.9836072352
C131	9.9122622127	-4.5677892195	8.7278071885
N132	9.0419343290	-4.6874068932	9.8065382243
C133	9.3536422365	-3.6157327774	7.9208508142
H134	9.6941957332	-3.2002224193	6.9881066493
C135	8.0041277147	-3.8512055495	9.6576737182
H136	7.1657914322	-3.7251593284	10.3253114188
N137	8.1854548664	-3.1924739593	8.5180658589
H138	7.4860600282	-2.5282849575	8.1084181020
C139	6.0985558496#	-4.2351691200#	-1.2221437475#
H140	5.3834490846#	-4.5684390086#	-1.9593380780#
H141	7.1094699709#	-4.1302109532#	-1.5886320672#
H142	5.7986810385	-3.2142022015	-0.9526943084
C143	6.0161738416	-5.1014645815	0.0545623087
H144	5.9473492542	-6.1663960040	-0.1874079101
H145	6.9214404799	-4.9469880681	0.6548910410
C146	4.8236866571	-4.6680896825	0.9146462589
O147	4.8585172469	-3.4979291947	1.3917926629
O148	3.8597734857	-5.4804158118	1.0974681418
C149	-1.8288114851#	-11.9864049815#	4.2486990958#
H150	-2.3586331862#	-12.7181588065#	3.6562799274#
H151	-2.3284821668#	-11.7833160253#	5.1842199164#
H152	-0.8468681301	-12.4065879806	4.5021307891
C153	-1.6647114435	-10.6500511357	3.4824031344
H154	-2.6040263654	-10.4264761033	2.9547822858
H155	-1.5133988273	-9.8401545936	4.2064314787
C156	-0.4939395267	-10.6379921222	2.4826470226
H157	0.4472081703	-10.5425059923	3.0415519616
H158	-0.4446005095	-11.6065615031	1.9653758048
C159	-0.5827133047	-9.5590589515	1.3838910595
H160	0.2434060383	-9.6881960336	0.6705408193
H161	-1.5034779383	-9.7303198337	0.8095304943
C162	-0.6218589312	-8.0847452219	1.8077911806
H163	-0.8995028853	-7.4703705649	0.9459393624
H164	-1.3514682890	-7.9050673575	2.6017815051
N165	0.6937604246	-7.5613569373	2.3058134187
H166	0.7421724824	-6.5249178459	2.2489245988
H167	0.8349099543	-7.8004033966	3.3297802340
H168	1.5086449222	-7.8968147613	1.7396303223
Cl169	0.8789808559	-7.9485241823	5.3288770190
O170	1.4862067142	-2.1194525810	2.8690119105
H171	2.6701333736	-1.1483593426	1.4581079820
O172	4.4175394759	-1.1213600179	4.1867640306
O173	7.0981865378	-1.9787548188	1.9220860646
H174	6.9562529145	-1.9760094831	2.8869142693
H175	6.3609805672	-2.5573637807	1.5952808977
O176	1.1322000305	1.5906839514	6.7298634386
H177	0.5337667079	0.9206889617	6.2858490236
H178	0.8474433259	1.5541542695	7.6574530198

O179	3.2596892347	3.2818459745	8.4746873370
H180	4.0493567781	3.4019713007	9.0417879095
H181	2.6236988721	2.8590561334	9.0792388882
O182	5.4067883115	2.6090424902	10.1478975694
H183	5.3910413569	1.7537662059	9.6692083025
H184	6.0209784266	2.4619549271	10.8942009008
H187	-0.7795981135	-0.4699754261	4.9883727324
O188	2.2637336549	-1.2574785724	5.6896675894
O189	1.4081409897	-4.7355224675	1.8663282574
H190	2.3759343009	-4.9212076666	1.6585057093
H191	1.0397611797	-4.4531894546	1.0130142500
O192	3.5771857307	-1.0234816467	1.1271267602
H193	3.9679927696	-1.9260488814	1.1574857543
O195	2.9751750808	-8.0285431907	0.7276817917
H196	3.4067463303	-7.1361290901	0.7364170114
H197	3.6470788721	-8.6382620027	1.0660923202
O198	-0.4989772807	-0.3558238598	5.9298618910
H199	0.0569825697	-1.1325167556	6.1042022320
H200	1.4927055600	-3.1068758519	2.7581358225
H201	9.1611042209	-5.3238127103	10.5851550907
O202	-0.9939655364	-1.0437564177	3.3436588489
H203	-0.1337324937	-1.4026727896	3.0250043221
H204	-1.4916540646	-0.7583162322	2.5662418035

The approximate O-O TS structure in **Figure 5.**

Energies: E= -5850.459109, solv= -0.130016, disp= -0.394821
The spins on Mn: 2.84, -3.41, 2.84, 2.94 ; O188-spin -0.54

Mn1	2.6528404345	-1.5150312197	4.0473386666
Mn2	4.6215000169	-1.4516360811	8.5717206141
Mn3	5.2924109351	-1.4701160952	5.6685647628
Mn4	6.6636846176	0.0840527395	7.4930598932
O5	3.8025038255	-1.2948809239	6.8081345518
O6	5.7678482672	0.2647409348	5.9660529670
O7	5.1388788798	0.3258045629	8.4521384525
O8	6.1379150684	-1.7574382765	7.4667189766
Ca9	3.5301930568	1.3587776322	6.8002690309
C10	0.6676532107#	1.9511745599#	1.7064590980#
H11	1.2251369033#	1.9059331478#	0.7825399340#
H12	-0.3243509455#	2.3687461364#	1.6143040339#
H13	0.4655849328	0.8945022510	1.9304969109
C14	1.4918297489	2.4932842612	2.8888656551
H15	0.8321670676	2.9088019133	3.6636435874
H16	2.1607986948	3.3123995071	2.6037097487
C17	2.3403787351	1.4571862990	3.6503258138
O18	3.2591371713	1.8603181221	4.4117551278
O19	1.9978226369	0.2330074276	3.4886441199
C20	1.0582650584#	1.1828949692#	13.4562169112#
H21	0.3702100002#	0.3601589986#	13.5824969933#
H22	0.7336609903#	2.1291440000#	13.8635909920#
H23	1.9539654891	0.9076326585	14.0326641850
C24	1.4471702669	1.2857013229	11.9629453201
H25	2.2329787771	2.0408249795	11.8313732325
H26	0.5898429806	1.6155077517	11.3639690097
C27	1.9490797110	-0.0703250177	11.4385835255
H28	1.1357164611	-0.8106424341	11.4653982950
H29	2.7289982043	-0.4717277528	12.0976036255
C30	2.5151831064	-0.1048524738	10.0227135111
O31	2.3426271520	0.7906113783	9.1875575512
O32	3.1946943799	-1.1995953051	9.7688459743

C33	2.7843440551#	-7.8606739733#	8.6258359289#
H34	2.5061953755	-8.8217099268	9.0755266356
H35	1.9466946514	-7.5382214643	8.0008249381
C36	4.0360829525	-8.0654332987	7.7593452900
O37	5.1742571467	-8.1682568526	8.2749924761
C38	3.0276292451	-6.8512456292	9.7880082440
H39	3.6795496383	-7.3275920180	10.5303648775
H40	2.0728305138	-6.6510925909	10.2826067110
C41	3.6496649554	-5.5485212448	9.3634105214
N42	4.8729811691	-5.5741476617	8.7134432974
H43	5.3565116811	-6.4607703653	8.5049106037
C44	3.3031072152	-4.2162461619	9.4126393652
H45	2.4404596115	-3.7371255946	9.8473908585
C46	5.1940504891	-4.3345964195	8.3369970375
H47	6.0185266873	-4.0941746297	7.6954503082
N48	4.2825803342	-3.4707440588	8.7683638902
N49	3.8387738220	-8.0088680024	6.4357378632
H50	2.8761632865	-7.9173406410	6.0756689737
C51	4.9175528857#	-7.8462328263#	5.4688790266#
H52	4.6133454783	-8.3470896223	4.5436456704
H53	5.8110181745	-8.3430283504	5.8530405686
C54	5.2321760953	-6.3453069225	5.1964090748
H55	6.0731038365	-6.2906000791	4.4922408367
H56	5.5864586012	-5.9009169612	6.1297894785
C57	4.0396272289	-5.5316482137	4.6567473881
H58	3.1016417981	-5.8357128392	5.1406602693
H59	3.8784904989	-5.7311292048	3.5908872462
C60	4.0910336963	-4.0112004822	4.7909745206
O61	3.0970227902	-3.4090180949	4.2653251958
O62	5.0486892842	-3.4475974357	5.4059670890
C63	9.1594979832#	0.1191439917#	11.4478219825#
H64	9.7884675130	-0.1551744472	10.5953527364
H65	9.7640617258	-0.0146858227	12.3552593628
C66	7.9255359810	-0.7757180843	11.5304082872
H67	7.2418091847	-0.4111776134	12.3030529200
H68	8.2080721328	-1.7949051501	11.8236239652
C69	7.1369631625	-0.8617288369	10.2284092119
O70	7.6454760446	-0.2629535078	9.2217549161
O71	6.0760439606	-1.5524879568	10.2301251748
C72	8.7808194073	1.5989321336	11.3646411556
O73	7.6701984319	2.0130826212	11.7130510174
N74	9.7759850317	2.4278408261	10.9603811888
H75	10.6070807607	2.0249651309	10.5479464492
C76	9.5676959609#	3.8587859857#	10.7651459676#
H77	10.4964012075	4.3890094965	10.9855011623
H78	8.7893533541	4.1796878073	11.4597451882
C79	9.1997948085	4.1376147285	9.2903583164
O80	10.0716394207	4.2831475215	8.4401579747
N81	7.8611644731	4.1758158171	9.0239141704
H82	7.2017937455	3.8593803298	9.7280149815
C83	7.3551094070	4.2216898435	7.6649650869
H84	6.6619927867	5.0573453932	7.5303081734
H85	8.2201809375	4.3626175590	7.0087164063
C86	6.6142744731	2.9342334845	7.2755126606
O87	5.4995502580	2.9841327310	6.7436068894
O88	7.2746435981	1.8493847526	7.5756918749
C89	11.5817469591#	-0.6171230366#	3.4809209274#
H90	12.4441860122#	-1.2180699743#	3.7283950196#
H91	11.8303760147#	0.3821390071#	3.1551920328#
H92	11.1236118844	-1.0954446598	2.6038772934
C93	10.5270436599	-0.4853271801	4.6031503783
H94	10.0210362814	0.4810666334	4.4923300652
H95	10.9962604829	-0.4550337696	5.5925298678
C96	9.4504855421	-1.5796746654	4.5495417287

H97	9.8463180520	-2.5431921886	4.9061235742
H98	9.1368485455	-1.7618144972	3.5168094670
C99	8.1900590447	-1.2659740558	5.3471849479
O100	7.1035430189	-1.7417681806	4.8638868376
O101	8.2965028116	-0.5914165610	6.4161979751
C102	10.8187337886#	4.2285347931#	-0.8150141992#
H103	11.5403700863#	3.4839291032#	-0.5131309511#
H104	11.2010829706#	5.2306319832#	-0.6882297790#
H105	10.6830313135	4.1018258360	-1.8965238608
C106	9.4611566808	3.9852062497	-0.1187759683
H107	9.0162720910	3.0709664551	-0.5367468926
H108	8.7682421844	4.7973099129	-0.3744315653
C109	9.5433831735	3.8638592565	1.4128601232
H110	10.0103845936	4.7696657118	1.8180455940
H111	10.2041127575	3.0329965794	1.6952900111
C112	8.1947228350	3.7038957416	2.1402814832
H113	7.4882964571	4.4619222117	1.7734571706
H114	8.3521208564	3.8837827366	3.2104186257
N115	7.6084990927	2.3621065139	1.9931010759
H116	7.9800518527	1.7536012336	1.2766042249
C117	6.4634036315	1.9632241917	2.5939764769
N118	5.8055107508	2.8044147747	3.4018408969
H119	4.9556445186	2.4923606648	3.8799317640
H120	6.1387106605	3.7370193428	3.5852965732
N121	5.9614688374	0.7453663162	2.3689553170
H122	6.5324058160	-0.0391777745	2.0096254625
H123	5.2325691246	0.4042700562	2.9965641914
C124	11.8424469947#	-5.3931179953#	7.2492950048#
H125	11.2521780061#	-5.9724400040#	6.5546989983#
H126	12.7964609992#	-5.8524470020#	7.4617229986#
H127	12.0237398250	-4.3943599960	6.8397842906
C128	11.1831854513	-5.3421430397	8.6403684968
H129	11.8967679298	-4.9077416083	9.3551347125
H130	11.0025402227	-6.3694033150	8.9845457588
C131	9.9097575242	-4.5732570277	8.7224746118
N132	9.0478208411	-4.6806521381	9.8087259800
C133	9.3428940480	-3.6355083813	7.9053015174
H134	9.6772262405	-3.2340971058	6.9648900199
C135	8.0078745642	-3.8480625993	9.6533789793
H136	7.1747193689	-3.7114232396	10.3254240700
N137	8.1786987306	-3.2042301861	8.5044031455
H138	7.4689845096	-2.5446859875	8.0931642074
C139	6.0985558331#	-4.2351690288#	-1.2221436595#
H140	5.3834491080#	-4.5684390408#	-1.9593380862#
H141	7.1094699592#	-4.1302109905#	-1.5886321093#
H142	5.8037730567	-3.2084208865	-0.9669469410
C143	5.9783255531	-5.0823859084	0.0649389522
H144	5.8777115317	-6.1478992342	-0.1626023190
H145	6.8757567745	-4.9464210307	0.6800444185
C146	4.7790012936	-4.5969614011	0.8855337464
O147	4.8900192825	-3.4314556384	1.3818657030
O148	3.7483184022	-5.3269244703	1.0016644571
C149	-1.8288114841#	-11.9864049840#	4.2486991030#
H150	-2.3586331820#	-12.7181588005#	3.6562799164#
H151	-2.3284821677#	-11.7833160192#	5.1842199147#
H152	-0.8454608696	-12.4043220212	4.5000099152
C153	-1.6717017177	-10.6471382034	3.4841087664
H154	-2.6067423083	-10.4397486958	2.9433714434
H155	-1.5501961255	-9.8335316699	4.2099430822
C156	-0.4874519905	-10.6094336805	2.4992882831
H157	0.4452128040	-10.4852505266	3.0664256365
H158	-0.4061721057	-11.5807035027	1.9915442916
C159	-0.5955765710	-9.5434999322	1.3891002573
H160	0.2300234798	-9.6686545473	0.6743314472

H161	-1.5139178403	-9.7403968538	0.8192043719
C162	-0.6583753938	-8.0626251061	1.7919949313
H163	-1.0027681348	-7.4739218974	0.9362567235
H164	-1.3512606246	-7.8896491147	2.6198057527
N165	0.6626996070	-7.4842779823	2.2100289883
H166	0.6767070810	-6.4466198804	2.1149596499
H167	0.8601400804	-7.6628152402	3.2345399783
H168	1.4658641314	-7.8187130867	1.6277795195
Cl169	0.9934491429	-7.5367942663	5.2377704151
O170	0.9880908109	-2.1257128759	3.5292536061
H171	2.1792174388	-2.0492385490	1.7362593186
O172	4.4440834107	-1.0663195395	4.1983632653
O173	7.0643538932	-1.7807071326	1.9599506724
H174	6.9913577168	-1.9196886494	2.9233435608
H175	6.3546129661	-2.3628194931	1.5972250775
O176	1.1324831976	1.8241477556	6.5952498640
H177	0.5703944702	0.9896014990	6.6868277288
H178	0.7878974694	2.4296795809	7.2702485285
O179	3.1722573101	3.2137915471	8.4895967719
H180	3.9838228288	3.4341900904	8.9881789661
H181	2.6633868801	2.6770645564	9.1280523434
O182	5.3843995247	2.6142905260	10.1528347705
H183	5.3640587730	1.7600357375	9.6726803167
H184	6.0020979717	2.4604455081	10.8947335002
H187	-0.7018183208	-0.5837022413	6.0310077062
O188	2.2456193946	-1.2052079427	5.7227452428
O189	1.2291957058	-4.6535260665	1.8159738435
H190	2.1915579911	-4.7985399935	1.5990343156
H191	0.8289958652	-4.3652521716	0.9797194380
O192	3.0611048430	-1.7400320413	2.0128218047
H193	3.7273071876	-2.4833530529	1.8082967062
O195	2.9509638991	-7.9137063582	0.6168423249
H196	3.3461490368	-7.0054629850	0.6405439864
H197	3.6366139319	-8.4960429177	0.9767749170
O198	-0.2039348695	-0.4338665124	6.8807133613
H199	0.5451561554	-1.0575537515	6.8139487004
H200	1.0106398578	-3.1013790890	3.4469568624
H201	9.1730612064	-5.3039415371	10.5969497090
O202	-1.1940306562	-0.7609814493	4.3757361700
H203	-0.4558681288	-1.3218224130	4.0298409785
H204	-1.0342139301	0.1005692898	3.9631126144
